# Cryopreservation of jaguar (*Panthera onca*) fibroblast-like cells in straws and cryovials: a comparison of rapid and slow-freezing protocols

**DOI:** 10.1590/1984-3143-AR2025-0096

**Published:** 2026-07-24

**Authors:** Sofia Regina Polizelle, Ian Figueiredo Navarezi, Maite Cardoso Coelho da Silva, Giulia Correia Vieira, Liandra Martiliano Gaeth, Larissa Schneider Brandão-Souza, Bianca Rodrigues Acacio, Pedro Nacib Jorge-Neto, Eliane Vianna da Costa e Silva, Thyara de Deco-Souza, Alexsandra Fernandes Pereira, Gediendson Ribeiro de Araujo

**Affiliations:** 1 Universidade Federal de Mato Grosso do Sul, Campo Grande, MS, Brasil; 2 Grupo de Pesquisa Reproduction for Conservation, Campo Grande, MS, Brasil; 3 Faculdade de Medicina Veterinária e Zootecnia, Universidade de São Paulo, São Paulo, SP, Brasil; 4 Laboratório de Biotecnologia Animal, Universidade Federal Rural do Semi-Árido, Mossoró, RN, Brasil

**Keywords:** big cats, biobanking, conservation, cryopreservation, felids

## Abstract

Somatic cells serve as essential genetic resources for biobanking. Traditionally, jaguar fibroblast-like cells are frozen in cryovials using slow-freezing protocols. However, this approach necessitates access to ultra-low temperature freezers and requires substantial storage capacity. This study aimed to evaluate the efficacy of a rapid-freezing protocol as a feasible alternative for the freezing of jaguar fibroblasts. Following cultivation to the third passage, cells were frozen using either slow-freezing (SF) or rapid-freezing (RF). For SF, cells were frozen in cryovials (-1 ºC/min), whereas RF involved rapid-freezing (-60 ºC/min) using nitrogen vapor and storage in 0.25 mL straws. Post-thaw, cells were re-cultured to assess recovery. Cell viability was assessed via light microscopy (Trypan blue), fluorescence microscopy (acridine orange + propidium iodide), and flow cytometry (Hoechst 33342 + propidium iodide) immediately post-thaw and upon reaching confluency (> 80%). Fresh culture viability assessed by Trypan blue, acridine orange, and flow cytometry was 98.2 ± 0.3%, 88.7 ± 3.9%, and 94.2 ± 2.2%, respectively. No significant differences in viability were observed between SF and RF immediately post-thaw or after subsequent cultivation. All three assays revealed significant differences (*p* < 0.05) between cells assessed immediately after thawing and those assessed at confluence. Upon reaching confluence, there was no significant difference (*p* > 0.05) between fresh and frozen-thawed cells. These findings indicate that RF in straws is a viable alternative to SF for jaguar fibroblast cryopreservation, offering optimized storage density for genetic resource banks.

## Introduction

The jaguar (*Panthera onca* Linnaeus, 1758), among the largest extant felids, has experienced a population decline attributed to anthropogenic factors such as hunting, habitat fragmentation, and wildlife-vehicle collision casualties. These factors contribute to genetic isolation and reduced genetic diversity (Alvarenga et al., 2025; Bogoni et al., 2023; Lorenzana et al., 2022; Meißner et al., 2025; Morato et al., 2014; Porfirio, 2019; Zenato Lazzari et al., 2025). In response to these challenges, various biotechnologies, including artificial insemination (Jorge-Neto et al., 2021; Mastromonaco and Songsasen, 2020), *in vitro* embryo production (Baldassare et al., 2015; Jorge-Neto et al., 2023), and somatic cell culture, have been utilized to preserve the genetic diversity of these populations (Deco-Souza et al., 2024; Mestre‐Citrinovitz et al., 2016). Furthermore, genetic banks have been established (Praxedes et al., 2018), in alignment with the One Conservation principle (Luczinski et al., 2023; Pizzutto et al., 2021). Somatic biopsies are readily collected, transported, and can be maintained for extended periods through vitrification. In conjunction with biobanks, cell lines can be derived from these cryopreserved tissues.

Somatic cells are advantageous genetic sources for biobanks because, unlike reproductive cells, they are easily collected and can be recovered post-mortem from animals of any age or sex. To achieve effective preservation, it is essential to implement protocols that ensure post-thaw viability while optimizing storage space (Praxedes et al., 2018; Urio, 2012). In jaguars, cell freezing utilizing slow-freezing protocols with isopropyl alcohol cooling in cryogenic tubes has demonstrated an average viability of 70% (Arantes et al., 2021; Mestre‐Citrinovitz et al., 2016; Oliveira et al., 2021). However, this method requires an ultra-low temperature freezer for the freezing process and demands substantial storage space.

Efforts to improve the efficacy and practicality of ultra freezer-independent somatic cell cryopreservation have progressed significantly. These include the vitrification of bovine fibroblasts (Gajda et al., 2007), freezing bovine fetal cells in straws using liquid nitrogen vapor (Liposki et al., 2018), and optimizing straw storage within cryogenic cylinders. Within this context, the primary objective of this study was to assess whether rapid-freezing in liquid nitrogen vapor, using 0.25 mL straws, is a feasible alternative for freezing jaguar somatic cells, thereby expanding biobanking capabilities for this iconic endangered species.

## Methods

The present study was conducted under authorization for scientific activities issued by SISBIO/ICMBio/MMA under no. 75762-1 and was approved by the Ethics Committee on Animal Use of the Federal University of Mato Grosso do Sul (CEUA/UFMS) under protocol No. 1.278/2023. Access to genetic heritage for all individuals was recorded in the Brazilian National System for the Management of Genetic Heritage and Associated Traditional Knowledge (SISGEN).

Unless otherwise specified, all reagents and media were sourced from Sigma-Aldrich (St. Louis, USA) and Gibco (Carlsbad, USA).

### Animals

This experiment utilized five jaguars from the scientific breeding center at the Jaguar Conservation Fund, located in Mineiros, Goiás, Brazil (17°54'00.5"S 53°00'21.7"W), as detailed in Table 1. For purposes of traceability and reference, these specimens were registered in Reprobank.org under the following unique identifiers: 4CGXQ-A0025, 4CGXQ-A0026, 4CGXQ-A0061, 4CGXQ-A0038, and 4CGXQ-A0016.

**Table 1:** Characterization of the jaguars (*Panthera onca*) utilized in the study, including sex, biome of origin, coat color, and estimated age.

**Animal Id**	**Study Id**	**Sex**	**Biome**	**Coat Color**	**Age**
4CGXQ-A0025	Ponca1	Male	Amazon	Non-melanistic	Adult (~ 4 y)
4CGXQ-A0026	Ponca2	Female	Amazon	Non-melanistic	Adult (~ 4 y)
4CGXQ-A0061	Ponca3	Female	Amazon	Melanistic	Adult (~ 10 y)
4CGXQ-A0038	Ponca4	Female	Cerrado	Non-melanistic	Adult (~ 4 y)
4CGXQ-A0016	Ponca5	Male	Cerrado	Non-melanistic	Adult (~ 5 y)

Anesthesia was induced using a combination of ketamine (5 mg/kg, im, Dopalen, Vetbrands, SP, Brazil) and medetomidine (0.1 mg/kg, im, Precision Pharmacy, CA, USA) delivered via anesthetic darts fired from a specialized rifle or blowpipe (Araujo et al., 2020). Following biological sample collection, yohimbine (0.4 mg/kg, im, Reset, Botupharma, SP, Brazil) was administered to reverse sedation (Jorge-Neto et al., 2020, 2026).

### Sample collection and storage

Following fur clipping and surface decontamination using 70% ethyl alcohol and chlorhexidine, a 2 cm^2^ ear fragment was aseptically excised from the marginal pinna of each animal using sterile surgical instruments. Subsequently, the wound edges were sealed with surgical adhesive, and a larvicidal topical repellent was applied. The biopsies were carefully placed into 2 mL cryogenic tubes containing Dulbecco's Modified Eagle Medium (DMEM), supplemented with 15% fetal bovine serum (FBS) and 5% antibiotic-antimycotic solution (Praxedes et al., 2019). Samples were transported to the laboratory in a portable refrigerator (TED Equipamentos, Cravinhos, São Paulo, Brazil) maintained at 4 °C and processed within twenty-four hours.

### Processing of samples, primary culture, and subcultures

Upon arrival, the ear fragments underwent a second fur clipping to remove residual hair. Subsequently, the fragments were rinsed twice with medium (DMEM + 15% FBS + 5% antibiotic-antimycotic solution) after a one-minute wash with 70% ethyl alcohol. To minimize contamination risk, the skin was carefully removed, and the cartilaginous tissue was washed with medium (DMEM + 15% FBS + 2% antibiotic-antimycotic solution) in a biosafety cabinet. The cartilage was then dissected into 0.5 x 0.5 cm fragments, targeting the perichondrium region to ensure a successful somatic cell outgrowth.

These tissue fragments were placed in 6-well plates with 1 mL of medium (DMEM + 15% FBS + 2% antibiotic-antimycotic solution) and incubated at 38.5 °C with 6% CO_2_. The culture medium was replaced every 24 hours. Upon reaching 80% confluence, cells were dissociated using 0.25% Trypsin-EDTA for subculture.

### Cryopreservation and thawing

During the third passage, when cells reached 70 to 80% confluence, freezing was conducted using two protocols: slow and rapid-freezing. First, the culture medium was aspirated, and the cells were rinsed twice with PBS. Trypsin (0.25% Trypsin-EDTA) was then added and incubated for 5 min. After confirming complete cell detachment, the trypsin was inactivated with culture medium. The suspension was centrifuged at 400 g for 5 min. Following supernatant removal, the cells were resuspended, and the concentration was adjusted to 1 x 10^6^ cells/mL. The cryoprotective medium consisted of DMEM containing 10% DMSO and 15% FBS, mixed in a 1:1 ratio (Karima et al., 2019).

### Slow-freezing

After adjusting the cell concentration, 1 mL of the suspension was transferred to cryogenic tubes. These tubes were then placed in a Mr. Frosty freezing container (Thermo Scientific Nalgene, Rochester, NY, USA) and stored in an ultra-low temperature freezer at -80 °C. The freezing rate was maintained at -1 °C/min for 12 h before the tubes were transferred to a liquid nitrogen tank (-196 °C). To thaw, the cryovials were equilibrated at 25 °C for 1 min and then immersed in a 37 °C water bath for 4 min. The contents were transferred to a tube containing culture medium and centrifuged to remove the cryoprotectant. After discarding the supernatant, the cell pellet was resuspended in 1 mL of culture medium. The remaining cells were seeded into a Petri dish and incubated, while an aliquot was reserved for viability analysis.

#### Rapid-freezing

The cryoprotectant-treated cell suspension was thoroughly mixed and loaded into 0.25 mL straws using a modified insulin syringe. Cells were positioned in the center of the straw, flanked by air columns. Subsequently, the straws were sealed with polyvinyl alcohol and equilibrated at 5 °C for 30 min. After this period, they were exposed to nitrogen vapor, positioned 4 cm above the liquid nitrogen surface for 5 min, resulting in a freezing rate of approximately -60 °C/min. Straws were then submerged in liquid nitrogen for a 15-day storage period (Liposki et al., 2018). To thaw, the straws were removed and immediately immersed in a 37 °C water bath for 20 s. Both ends of the straws were cut, and the cell suspension was transferred to centrifuge tubes containing 1 mL of culture medium. The mixture was centrifuged at 400 g for 5 min to eliminate the cryoprotectant. Following removal of the supernatant, the cell pellet was resuspended. An aliquot was extracted for viability assessment, while the remaining cells were seeded for further cultivation.

### Morphological analysis

Morphological assessment of the somatic cells, evaluating cell shape and cytoplasmic extensions, was conducted using an inverted microscope (Nikon TiU, Tokyo, Japan) according to the methodology described by Lira et al. (2022).

### Cell viability analysis

Cell viability was determined using three distinct techniques: Trypan blue exclusion for bright-field microscopy, acridine orange/propidium iodide (AO/PI) dual staining for fluorescence microscopy, and Hoechst 33342 combined with PI for flow cytometry. Cell viability was assessed at three experimental stages: before freezing (fresh), immediately after thawing, and upon reaching confluence. These methods were selected to monitor the percentage of viable cells throughout the freezing and recovery processes.

#### Trypan blue

For the Trypan blue exclusion assay, 20 μL of cell suspension and 20 μL of 0.4% Trypan blue were combined in conical tubes. Viable cells, which excluded the dye, were counted using a Neubauer chamber. The percentage of viable cells was calculated by dividing the number of unstained cells by the total cell count (Strober, 1997).

#### Acridine orange and propidium iodide

For the AO/PI assay, 20 μL of cell suspension, 20 μL of acridine orange (100 μg/mL), and 4 μL of PI (2 mg/mL) were combined in a light-protected conical tube. An aliquot of this mixture was then placed on a slide and covered with a coverslip for analysis using an inverted fluorescence microscope (Nikon, Ti2, Tokyo, Japan). One hundred cells per sample were counted and categorized as viable (uniform green nuclei), apoptotic (orange-stained), or necrotic (red nuclei).

#### Hoechst 33342 and propidium iodide

Flow cytometric analysis (CytoFLEX, Beckman Coulter) was performed using Hoechst 33342 and PI. In this assay, 40 μL of cell suspension was incubated with 20 μL of Hoechst 33342 (40 μg/mL) and 4 μL of PI (2 mg/mL) for 15 min. The percentage of viable cells was determined by the exclusion of PI staining.

### Statistical analysis

Data normality was assessed using the Shapiro-Wilk test, and viability data were arcsine-transformed ($y=\text{arcsin}\sqrt{x/100}$) prior to analysis. Subsequently, an analysis of variance (ANOVA) was performed to evaluate the effects of treatment, experimental stage (fresh, thawed, and confluent), and their interaction. A second model evaluated the effects of treatment (slow *vs*. rapid-freezing), analytical method (Trypan blue, AO/PI, and flow cytometry), and their interaction.

Means were compared using Student’s t-test where appropriate, and interaction means were adjusted and compared using the Tukey test. Differences were considered statistically significant at *p* ≤ 0.05. All analyses were performed using SAS software, and data were expressed as mean ± standard error.

## Results

Although somatic cell membrane integrity was significantly reduced immediately following thawing, no significant differences (*p* > 0.05) were observed between confluent cells derived from frozen samples and those from fresh cultures (Table 2). However, comparisons between the thawing and confluence time points revealed that Trypan blue and acridine orange detected variations in membrane integrity.

**Table 2:** Effect of slow and rapid-freezing on the membrane integrity of jaguar somatic cells at different experimental stages.

**Method**	**Fresh**	**Slow-freezing**		**Rapid-freezing**	
		**PT**	**CONF**	**PT**	**CONF**
**TB**	98.2 ± 0.3^a^	86.7 ± 3.7^b^	99.2 ± 0.5^a^	83.8 ± 4.5^b^	99.6 ± 0.1^a^
**AO/PI**	88.7 ± 3.9^a,b^	79.4 ± 1.9^b,c,d^	92.0 ± 1.4^a^	73.0 ± 6.7^d^	87.8 ± 2.7^a,c^
**Flow**	94.2 ± 2.2^a^	-	94.9 ± 0.6^a^	-	92.7 ± 1.7^a^

TB – Trypan blue; AO/PI – Acridine orange and Propidium Iodide; Flow – Flow cytometry (PI and Hoechst 33342); PT – Post-thaw analysis; CONF – Analysis at confluence. Different letters within the same row (a, b, c, d) indicate a statistically significant difference (*p* < 0.05).

**Figure 1:**
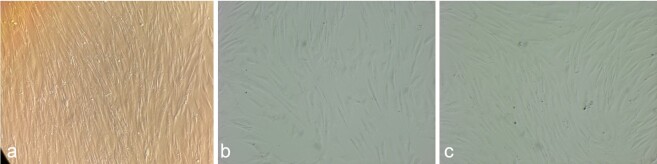
Jaguar somatic cell morphology at 80–90% confluence following specific treatments: (a) fresh culture, (b) slow-freezing, and (c) rapid-freezing (magnification ×100).

Morphological evaluation upon reaching confluence showed that cells from both freezing methods exhibited typical morphology, characterized by a fusiform shape, elongated nuclei, and prominent cytoplasmic extensions. This phenotype was consistent with observations in fresh cultures (Figure 1).

**Figure 2:**
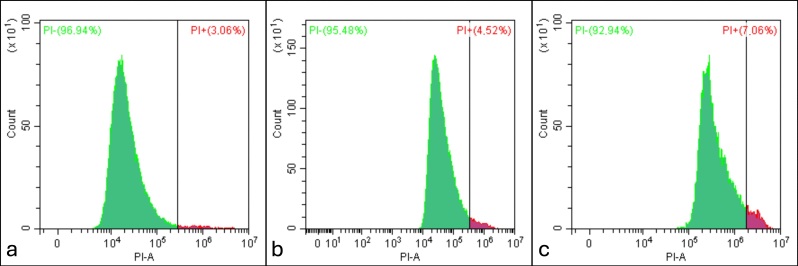
Representative flow cytometry histograms of jaguar somatic cells dual-stained with Hoechst 33342 and propidium iodide (PI): (a) fresh culture, (b) slow-frozen, and (c) rapid-frozen treatments. The y-axis represents the cell count, while the x-axis indicates PI fluorescence intensity. Viable cells (PI-negative) are characterized by low fluorescence intensity, whereas non-viable cells (PI-positive) exhibit high fluorescence intensity. The numerical values in the upper panels display the percentages of viable and non-viable populations.

Comparing the diagnostic methods for cell damage, Trypan blue staining indicated significantly higher membrane damage (p<0.05) in thawed cells relative to flow cytometry using fluorescent probes (Figure 2). viability results obtained from fluorescent staining (AO/PI and flow cytometry) were comparable (p>0.05) across all treatments (Table 3).

**Table 3:** Comparative analysis of cell viability and membrane integrity using distinct staining methods in jaguar somatic cells at the confluence stage.

**Treatment**	**Viability assessment method**		
	**AO/PI**	**TB**	**Flow**
**Fresh**	88.7 ± 3.9^b^	98.2 ± 0.4^a^	94.2 ± 2.2^a,b^
**Slow-freezing**	92.0 ± 1.4^a,b^	86.8 ± 3.7^b^	94.9 ± 0.6^a^
**Rapid-freezing**	87.8 ± 2.7^a,b^	83.8 ± 4.6^b^	92.7 ± 1.7^a^

TB – Trypan blue; AO/PI – Acridine orange and Propidium Iodide; Flow – Flow cytometry. Different letters within the same row (a, b) indicate a statistically significant difference (*p* < 0.05).

## Discussion

The present study aimed to identify the most effective protocol for the cryopreservation of cells derived from jaguar ear cartilage, with the goal of establishing a practical and standardized approach. Both slow-freezing—utilizing a Mr. Frosty device at a freezing rate of -1 °C/min to -80 °C—and rapid-freezing—achieved in nitrogen vapor at approximately -60 °C/min—were evaluated for their ability to maintain post-thaw membrane integrity and cell viability.

While slow-freezing has traditionally been the standard method, yielding average survival rates of 70 to 80% in jaguars when assessed by Trypan blue (Mestre‐Citrinovitz et al., 2016; Oliveira et al., 2021; Silva et al., 2021), it requires an ultra-low temperature freezer and occupies more storage space due to the use of cryogenic tubes. Conversely, in the current study, the average viability rates obtained following rapid-freezing using acridine orange, Trypan blue, and flow cytometry were 87.8% ± 2.7, 83.8% ± 4.5, and 92.7% ± 1.7, respectively. These results surpass the rates reported in previous studies utilizing slow-freezing methods (Arantes et al., 2021; Mestre‐Citrinovitz et al., 2016; Oliveira et al., 2021). Furthermore, freezing in liquid nitrogen vapor offers the distinct advantage of utilizing 0.25 or 0.5 mL straws, effectively optimizing cryopreservation storage density within liquid nitrogen tanks, as previously demonstrated in bovine somatic cells (Cetinkaya and Arat, 2011; Pacheco et al., 2022).

Throughout the cooling and freezing process, significant changes occur within the plasma membrane architecture, accompanied by the potential for ice crystal formation and fluctuations in intra- and extracellular osmolarity. These biophysical events can have a substantial impact on post-thaw cell viability. Slower freezing rates lead to cellular dehydration, resulting in increased intracellular solute concentrations. This process helps prevent lethal intracellular ice formation (IIF), a phenomenon driven by the “solute effect”. Conversely, excessively rapid-freezing rates can induce lethal IIF, leading to irreversible mechanical and structural damage (Elliott et al., 2017). To mitigate these effects, the thawing kinetics must be carefully managed: slowly frozen cells must undergo a gradual rewarming process, while rapidly frozen cells must be swiftly thawed to prevent ice recrystallization (Gao and Critser, 2000).

Regarding somatic cell cryopreservation in felids, including jaguars, the predominant practice has involved slow-freezing rates achieved through programmable freezers or passive cooling devices (Guan et al., 2010; Mestre‐Citrinovitz et al., 2016; Song et al., 2007). The present study compared the efficacy of the Mr. Frosty device against nitrogen vapor freezing in straws to assess different freezing rates, which may involve distinct cryoinjury mechanisms. However, the efficacy of both methods in preserving cellular integrity and post-thaw viability was comparable. The average cell viability rates were 86.7% ± 3.7 and 79.4% ± 1.9 for slow-freezing, compared to 83.8% ± 4.5 and 73.0% ± 6.7 for rapid-freezing, as determined by Trypan blue and acridine orange, respectively.

Another factor inherently linked to cryoinjury mechanisms is the choice of storage container. Large-diameter containers, such as cryovials, exhibit greater thermal gradients between the periphery and the center compared to smaller containers like straws, which facilitate a more uniform freezing rate (Araújo et al., 2013; Hunt, 2019; Meneghel et al., 2020). However, it is noteworthy that 0.25 mL straws are more susceptible to temperature fluctuations during handling and require more precise manipulation. Consequently, the advantages of space optimization offered by straws must be weighed against their heightened sensitivity during handling.

Nevertheless, prior investigations involving bovine somatic cells (Urio, 2012) did not reveal any significant differences between cells frozen in 0.5 mL and 0.25 mL straws. Furthermore, Liposki et al. (2018) reported a somatic cell viability of 89.8% when freezing bovine fetal cells in 0.25 mL straws using the Trypan blue exclusion assay. In cattle, Forell et al. (2008) and Rochetti et al. (2010) demonstrated the effectiveness of 0.5 mL straws for freezing somatic cells intended for cloning techniques, highlighting their capacity to preserve twice the volume of 0.25 mL straws.

To protect cells against cryoinjury during the freezing process, it is essential to employ cryoprotective agents (CPAs) that act both intra- and extracellularly to modulate membrane dynamics and intracellular components. Ideal CPAs should exhibit minimal toxicity, be biologically compatible, and maintain cell viability, thereby reducing apoptotic cell death during cryoprocessing (Jang et al., 2017). Extracellular cryoprotectants, such as sucrose and trehalose, are high-molecular-weight compounds consisting of sugars, lipoproteins, and certain amino acids (Gonzalez, 2004; Janz et al., 2012; Mattos et al., 2023). Dimethyl sulfoxide (DMSO), ethylene glycol, and glycerol are the most common intracellular CPAs (Sreter et al., 2022). Notably, the low-molecular-weight DMSO binds to water molecules within the intracellular environment, inhibiting the formation of lethal ice crystals (Cetinkaya and Arat, 2011; León-Quinto et al., 2014). Moreover, DMSO is pivotal for stabilizing cellular proteins and maintaining electrolyte balance across the cell membrane (Liu et al., 2010).

In some instances, a combination of intracellular and extracellular cryoprotectants is employed to mitigate osmotic stress (Schneider and Mazur, 1984). However, in experiments involving jaguar cells—which resemble fibroblasts morphologically—subjected to slow-freezing, sucrose provided no additional protective benefits (Oliveira et al., 2021). Consequently, the present study utilized a 10% (v/v) concentration of DMSO, confirming its efficacy in both proposed freezing methods.

Both AO and Trypan blue analyses indicated an increase in cell viability following cultivation relative to the immediate post-thaw state. This phenomenon conforms to observations in other jaguar studies, where viability recovered post-culture to levels similar to fresh cells (97.8% ± 1.1 *vs*. 95.7% ± 0.3, respectively; *p* < 0.05) (Oliveira et al., 2021). This recovery can be attributed to the removal of non-viable cells in suspension during medium changes, effectively eliminating debris while allowing healthy cells to adhere and proliferate. While some authors (Silva et al., 2021) investigated freezing effects at the confluence stage, others analyzed cells immediately after thawing (Borges et al., 2020; Jenuit et al., 2021; Magalhães et al., 2017; Praxedes et al., 2021). In the present study, we observed that while viability fluctuates acutely after thawing, it returns to levels comparable to fresh cells after cultivation. Because the effects of cryoinjury may be masked after cell culture, it is critical to prioritize the evaluation of freezing treatments immediately after thawing.

Cell viability assessments based on morphology, physiological state, or membrane permeability can be validated through fluorescent probes (Johnson et al., 2013). Trypan blue exclusion is extensively employed as it penetrates damaged membranes, allowing for the differentiation of intact and compromised cells (Strober, 1997). This assay is independent of enzymatic activity, as its effectiveness is solely dependent on membrane integrity. However, Trypan blue is susceptible to artifacts that may interfere with the accuracy of the analysis (Mascotti et al., 2000).

The use of fluorescent probes like PI in microscopy or flow cytometry is another prevalent technique. Similar to Trypan blue, PI stains cells with compromised membranes, but the application is generally more expensive (Lebeau et al., 2019). Conversely, AO emits green fluorescence upon binding to the nucleic acids of viable cells. In combination with PI, it exhibits orange or red fluorescence depending on the degree of cell injury. This method is efficient, rapid, and deemed superior to Trypan blue because it facilitates additional differentiation between viable, apoptotic, and necrotic cells (Johnson et al., 2013).

In the present study, AO staining and flow cytometry using PI/Hoechst 33342 showed no significant differences in fresh cell viability (*p* > 0.05). Nevertheless, discrepancies were observed between these techniques and the Trypan blue assay. Trypan blue revealed a lower percentage of viable cells in thawed samples compared to flow cytometry. This discrepancy can be attributed to the sample size analyzed via Trypan blue, as well as the presence of dye precipitates, which can generate artifacts and lead to false positives, thereby overestimating the non-viable population. Kummrow et al. (2013) highlighted the challenges in detecting unstained cells, which often results in population overestimation when using Trypan blue. They suggest that to obtain more precise results, a larger cell count (>2500 cells for a statistical uncertainty of 2%) should be analyzed. Additionally, flow cytometry offers greater objectivity compared to manual microscopy, as the material is processed automatically, minimizing operator bias.

Furthermore, Kummrow et al. (2013) emphasized the ability of flow cytometry to perform rapid, multi-parametric, and quantitative analyses, whereas microscopy is more suitable for individual cell examinations. Flow cytometry and fluorescence microscopy produced similar results in the current study, as both approaches utilize comparable principles and primarily differ in the number of events evaluated. Consequently, the selection of the analytical method may depend on equipment availability and the specific requirements of the study.

## Conclusion

In conclusion, our findings demonstrate that both slow and rapid-freezing protocols are effective for the freezing of jaguar somatic cells, preserving both viability and proliferative capacity. However, when optimizing storage within liquid nitrogen cylinders, 0.25 mL plastic straws are superior to cryogenic tubes as they significantly increase storage density and biobank capacity. To accurately evaluate freezing efficacy, it is imperative to perform assessments immediately post-thaw, as the selective elimination of non-viable cells during medium replacement can mask the true extent of cryoinjury if analysis is delayed until confluence. By ensuring the long-term availability of high-quality genetic material, these results provide a foundational resource for future reproductive biotechnologies and the genetic management of jaguar populations. Furthermore, this streamlined approach has the potential to be adapted for the conservation of other endangered felids and non-domestic species, aligning with global “One Conservation” efforts.

## Data Availability

Research data is only available upon request.
